# New distributional data on aquatic and semiaquatic bugs (Hemiptera: Heteroptera: Gerromorpha & Nepomorpha) from South America

**DOI:** 10.3897/BDJ.3.e4913

**Published:** 2015-04-14

**Authors:** Isabelle R S Cordeiro, Felipe F F Moreira

**Affiliations:** ‡ Fundação Oswaldo Cruz, Laboratório Nacional e Internacional de Referência em Taxonomia de Triatomíneos/IOC, Rio de Janeiro, Brazil

**Keywords:** Aquatic insects, new records, Neotropical Region, Brazil, Peru.

## Abstract

**Background:**

Water bugs in general play an important role in freshwater ecosystems, and knowledge about them is essential for the study of water biology and the proper management of aquatic habitats. The Neotropical fauna is relatively well known, but the existence of large under-collected areas makes taxonomic and faunistic studies concerning the aquatic and semiaquatic bugs from tropical America urgent.

**New information:**

Distributional information is presented for thirty-eight species of Gerromorpha and five Nepomorpha, including first records from the Brazilian states of Bahia (*Mesovelia amoena*), Ceará (*Limnogonus profugus* and *Rhagovelia whitei*), Espírito Santo (*R. lucida*), Goiás (*Halobatopsis platensis* and *R. zela*), Mato Grosso (*Rheumatobates bonariensis*), Pará (*Nerthra terrestris*), Paraná (*H. spiniventris*, *Hydrometra fruhstorferi* and *R. janeira*), Piauí (*Microvelia ayacuchana*, *M. pulchella*, *Neogerris lubricus* and *Platyvelia brachialis*), Rio de Janeiro (*Martarega bentoi*) and São Paulo (*Rheumatobates minutus flavidus*); and the Peruvian region of Madre de Dios (*Rhagovelia fontanalis*).

## Introduction

Heteroptera (Insecta: Hemiptera), or true bugs, is the most diverse group of insects with incomplete metamorphosis, with about 38,000 described species (Ribeiro et al. 2014). This suborder comprises seven infraorders, of which Gerromorpha, Nepomorpha and Leptopodomorpha are associated with aquatic habitats (Rafael et al. 2012).

Aquatic Heteroptera can be found in all continents, except Antarctica, and are more diverse in tropical climates. The Neotropical region is the richest in terms of described species, with more than 1,200 distributed in 18 families (Polhemus and Polhemus 2008). Six families, 43 genera and 380 species of Gerromorpha, and ten families, 39 genera and 510 species of Nepomorpha are known from South America, according to the latest published information (Mazzucconi et al. 2009).

The current study presents new data concerning the geographical distribution of Gerromorpha and Nepomorpha from South America, including first species records from several Brazilian states and a Peruvian region.

## Materials and methods

The new information provided is based on material desposited in the Coleção Entomológica Professor José Alfredo Pinheiro Dutra, Departamento de Zoologia, Instituto de Biologia, Universidade Federal do Rio de Janeiro, Rio de Janeiro, Brazil (DZRJ) and the Laboratório de Estudos Subterrâneos, Universidade Federal de São Carlos, São Carlos, Brazil (LES). The known geographical distribution of each species is provided according to Moreira (2015). New records are followed by an exclamation mark. Abbreviations of Brazilian states follow the official standard (IBGE 2015).

## Taxon treatments

### Brachymetra albinervis albinervis

#### Materials

- **Type status:** Other material. **Occurrence:** individualCount: 3; sex: 1 apterous male, 2 apterous females; **Taxon:** genus: Brachymetra; specificEpithet: albinervis; infraspecificEpithet: albinervis; **Location:** continent: South America; country: Brazil; stateProvince: Rio de Janeiro; municipality: Rio de Janeiro; locality: Jacarepaguá, Taquara, Estrada Pau da Fome, Parque Estadual da Pedra Branca, Rio Manuel Justino; decimalLatitude: -22.9365; decimalLongitude: -43.4447; **Identification:** identifiedBy: I.R.S. Cordeiro; **Event:** year: 2013; month: 5; day: 26; eventRemarks: I.R.S. Cordeiro, F.A.C. Silva, F.F. Silva, L. Chaves & A. Ferreira col.; **Record Level:** type: PhysicalObject; institutionCode: DZRJ; basisOfRecord: PreservedSpecimen
- **Type status:** Other material. **Occurrence:** individualCount: 2; sex: 1 apterous male, 1 apterous female; **Taxon:** genus: Brachymetra; specificEpithet: albinervis; infraspecificEpithet: albinervis; **Location:** continent: South America; country: Brazil; stateProvince: Rio de Janeiro; municipality: Rio de Janeiro; locality: Jacarepaguá, Taquara, Estrada Pau da Fome, Parque Estadual da Pedra Branca, Rio Figueira; decimalLatitude: -22.9327; decimalLongitude: -43.438; **Identification:** identifiedBy: I.R.S. Cordeiro; **Event:** year: 2013; month: 6; day: 8; eventRemarks: Cordeiro, F.A.C. Silva, F.F. Silva & L. Chaves col.; **Record Level:** type: PhysicalObject; institutionCode: DZRJ; basisOfRecord: PreservedSpecimen
- **Type status:** Other material. **Occurrence:** individualCount: 12; sex: 2 apterous males, 10 apterous females; **Taxon:** genus: Brachymetra; specificEpithet: albinervis; infraspecificEpithet: albinervis; **Location:** continent: South America; country: Brazil; stateProvince: Rio de Janeiro; municipality: Rio de Janeiro; locality: Jacarepaguá, Taquara, Estrada Pau da Fome, Parque Estadual da Pedra Branca, Rio do Quilombo; decimalLatitude: -22.9323; decimalLongitude: -43.4358; **Identification:** identifiedBy: I.R.S. Cordeiro; **Event:** year: 2013; month: 6; day: 16; eventRemarks: I.R.S. Cordeiro & F.A.C. Silva col.; **Record Level:** type: PhysicalObject; institutionCode: DZRJ; basisOfRecord: PreservedSpecimen

#### Distribution

Guatemala, Honduras, Dominica, Martinique, St. Lucia, St. Vincent & Grenadines, Colombia, Grenada, Venezuela, Trinidad & Tobago, Panama, Suriname, Brazil, Ecuador, Peru, Paraguay.

**Distribution in Brazil:** PA, AM, CE, MT, BA, MG, SP, RJ.

### Brachymetra furva

#### Materials

- **Type status:** Other material. **Occurrence:** individualCount: 2; sex: 1 apterous male, 1 apterous female; **Taxon:** genus: Brachymetra; specificEpithet: furva; **Location:** continent: South America; country: Brazil; stateProvince: Rio de Janeiro; municipality: Rio de Janeiro; locality: Jacarepaguá, Taquara, Estrada Pau da Fome, Parque Estadual da Pedra Branca, Rio Manuel Justino; decimalLatitude: -22.9365; decimalLongitude: -43.4447; **Identification:** identifiedBy: I.R.S. Cordeiro; **Event:** year: 2012; month: 11; day: 19; eventRemarks: I.R.S. Cordeiro, F.A.C. Silva & L.Chaves col.; **Record Level:** type: PhysicalObject; institutionCode: DZRJ; basisOfRecord: PreservedSpecimen
- **Type status:** Other material. **Occurrence:** individualCount: 14; sex: 7 apterous males, 7 apterous females; **Taxon:** genus: Brachymetra; specificEpithet: furva; **Location:** continent: South America; country: Brazil; stateProvince: Rio de Janeiro; municipality: Rio de Janeiro; locality: Jacarepaguá, Taquara, Estrada Pau da Fome, Parque Estadual da Pedra Branca, Rio Manuel Justino; decimalLatitude: -22.9365; decimalLongitude: -43.4447; **Identification:** identifiedBy: I.R.S. Cordeiro; **Event:** year: 2013; month: 9; day: 7; eventRemarks: I.R.S. Cordeiro, F.A.C. Silva, F.F.Silva, L.Chaves & A. Ferreira col.; **Record Level:** type: PhysicalObject; institutionCode: DZRJ; basisOfRecord: PreservedSpecimen
- **Type status:** Other material. **Occurrence:** individualCount: 6; sex: 4 apterous males, 2 apterous females; **Taxon:** genus: Brachymetra; specificEpithet: furva; **Location:** continent: South America; country: Brazil; stateProvince: Rio de Janeiro; municipality: Rio de Janeiro; locality: Jacarepaguá, Taquara, Estrada Pau da Fome, Parque Estadual da Pedra Branca, Rio Figueira; decimalLatitude: -22.9327; decimalLongitude: -43.4380; **Identification:** identifiedBy: I.R.S. Cordeiro; **Event:** year: 2013; month: 8; day: 19; eventRemarks: I.R.S. Cordeiro, F.A.C. Silva, F.F.Silva, L.Chaves & A. Ferreira col.; **Record Level:** type: PhysicalObject; institutionCode: DZRJ; basisOfRecord: PreservedSpecimen
- **Type status:** Other material. **Occurrence:** individualCount: 27; sex: 10 apterous males, 17 apterous females; **Taxon:** genus: Brachymetra; specificEpithet: furva; **Location:** continent: South America; country: Brazil; stateProvince: Rio de Janeiro; municipality: Rio de Janeiro; locality: Jacarepaguá, Taquara, Estrada Pau da Fome, Parque Estadual da Pedra Branca, Rio do Quilombo; decimalLatitude: -22.9323; decimalLongitude: -43.4358; **Identification:** identifiedBy: I.R.S. Cordeiro; **Event:** year: 2013; month: 8; day: 24; eventRemarks: I.R.S. Cordeiro & F.A.C. Silva col.; **Record Level:** type: PhysicalObject; institutionCode: DZRJ; basisOfRecord: PreservedSpecimen
- **Type status:** Other material. **Occurrence:** individualCount: 9; sex: 5 apterous males, 2 macropterous males, 2 apterous females; **Taxon:** genus: Brachymetra; specificEpithet: furva; **Location:** continent: South America; country: Brazil; stateProvince: Rio de Janeiro; municipality: Rio de Janeiro; locality: Jacarepaguá, Taquara, Estrada Pau da Fome, Parque Estadual da Pedra Branca, Rio Grande; decimalLatitude: -22.9320; decimalLongitude: -43.4452; **Identification:** identifiedBy: I.R.S. Cordeiro; **Event:** year: 2014; month: 4; day: 19; eventRemarks: I.R.S. Cordeiro, F.A.C. Silva, F.F.Silva, L.Chaves, A. Ferreira, L.S. Barbosa, D.M.C. Santos & L.B. Pena Forte col.; **Record Level:** type: PhysicalObject; institutionCode: DZRJ; basisOfRecord: PreservedSpecimen

#### Distribution

Brazil, Argentina.

**Distribution in Brazil:** MA, PI, RN, MT, BA, MG, MS, RJ, PR.

### Halobatopsis platensis

#### Materials

- **Type status:** Other material. **Occurrence:** individualCount: 1; sex: 1 apterous male; **Taxon:** genus: Halobatopsis; specificEpithet: platensis; **Location:** continent: South America; country: Brazil; stateProvince: Goiás; municipality: Cristalina; decimalLatitude: -16.730222; decimalLongitude: -47.569056; **Identification:** identifiedBy: F.F.F. Moreira; **Event:** year: 2013; month: 3; day: 19; eventRemarks: D.M. Takiya & A.P.M. Santos col.; **Record Level:** type: PhysicalObject; institutionCode: DZRJ; basisOfRecord: PreservedSpecimen
- **Type status:** Other material. **Occurrence:** individualCount: 5; sex: 5 apterous females; **Taxon:** genus: Halobatopsis; specificEpithet: platensis; **Location:** continent: South America; country: Brazil; stateProvince: Goiás; municipality: Alto Paraíso de Goiás; decimalLatitude: -14.160972; decimalLongitude: -47.594167; **Identification:** identifiedBy: F.F.F. Moreira; **Event:** year: 2013; month: 3; day: 21; eventRemarks: D.M. Takiya & A.P.M. Santos col.; **Record Level:** type: PhysicalObject; institutionCode: DZRJ; basisOfRecord: PreservedSpecimen
- **Type status:** Other material. **Occurrence:** individualCount: 2; sex: 1 apterous male, 1 apterous female; **Taxon:** genus: Halobatopsis; specificEpithet: platensis; **Location:** continent: South America; country: Brazil; stateProvince: Goiás; municipality: Alto Paraíso de Goiás / São João da Aliança; decimalLatitude: -14.144194; decimalLongitude: -47.342667; **Identification:** identifiedBy: F.F.F. Moreira; **Event:** year: 2013; month: 3; day: 22; eventRemarks: D.M. Takiya & A.P.M. Santos col.; **Record Level:** type: PhysicalObject; institutionCode: DZRJ; basisOfRecord: PreservedSpecimen

#### Distribution

Brazil, Paraguay, Argentina, Uruguay.

**Distribution in Brazil:** PI, MT, BA, GO!, MG, DF, MS, SP, RJ, PR, RS.

### Halobatopsis spiniventris

#### Materials

- **Type status:** Other material. **Occurrence:** individualCount: 10; sex: 7 apterous males, 3 apterous females; **Taxon:** genus: Halobatopsis; specificEpithet: spiniventris; **Location:** continent: South America; country: Brazil; stateProvince: Paraná; municipality: Céu Azul; locality: Parque Nacional do Iguaçu, Rio Azul (3ª ordem); decimalLatitude: -25.1558; decimalLongitude: -53.7923; **Identification:** identifiedBy: F.F.F. Moreira; **Event:** year: 2012; month: 9; day: 6; eventRemarks: F. Capistrano col.; **Record Level:** type: PhysicalObject; institutionCode: DZRJ; basisOfRecord: PreservedSpecimen
- **Type status:** Other material. **Occurrence:** individualCount: 10; sex: 3 apterous males, 7 apterous females; **Taxon:** genus: Halobatopsis; specificEpithet: spiniventris; **Location:** continent: South America; country: Brazil; stateProvince: Paraná; municipality: Céu Azul; locality: Parque Nacional do Iguaçu, Rio Azul (3ª ordem); decimalLatitude: -25.1558; decimalLongitude: -53.7923; **Identification:** identifiedBy: F.F.F. Moreira; **Event:** year: 2012; month: 9; day: 6; eventRemarks: J. F. Barbosa col.; **Record Level:** type: PhysicalObject; institutionCode: DZRJ; basisOfRecord: PreservedSpecimen
- **Type status:** Other material. **Occurrence:** individualCount: 2; sex: 2 apterous males; **Taxon:** genus: Halobatopsis; specificEpithet: spiniventris; **Location:** continent: South America; country: Brazil; stateProvince: Paraná; municipality: Céu Azul; locality: Parque Nacional do Iguaçu, Rio Manuel Gomes; decimalLatitude: -25.1586; decimalLongitude: -53.8331; **Identification:** identifiedBy: F.F.F. Moreira; **Event:** year: 2012; month: 9; day: 7; eventRemarks: J. F. Barbosa col.; **Record Level:** type: PhysicalObject; institutionCode: DZRJ; basisOfRecord: PreservedSpecimen

#### Distribution

Brazil, Argentina.

**Distribution in Brazil:** SP, PR!, SC, RS.

### Limnogonus profugus

#### Materials

- **Type status:** Other material. **Occurrence:** individualCount: 1; sex: 1 macropterous female; **Taxon:** genus: Limnogonus; specificEpithet: profugus; **Location:** continent: South America; country: Brazil; stateProvince: Ceará; locality: Parque Nacional Ubajara, Rio das Minas, Trilha Araticum; **Identification:** identifiedBy: F.F.F. Moreira; **Event:** year: 2013; month: 2; day: 14; eventRemarks: A.P.M. Santos col.; **Record Level:** type: PhysicalObject; institutionCode: DZRJ; basisOfRecord: PreservedSpecimen

#### Distribution

Brazil, Peru, Paraguay, Argentina.

**Distribution in Brazil:** CE!, PB, PE, MT, GO, MG, MS, SP, RJ.

### Neogerris lubricus

#### Materials

- **Type status:** Other material. **Occurrence:** individualCount: 1; sex: 1 macropterous male; **Taxon:** genus: Neogerris; specificEpithet: lubricus; **Location:** continent: South America; country: Brazil; stateProvince: Piauí; municipality: Piracuruca; locality: Parque Nacional de Sete Cidades, Cachoeira do Riachão; verbatimElevation: 171 m; decimalLatitude: -4.1078; decimalLongitude: -41.6736; **Identification:** identifiedBy: F.F.F. Moreira; **Event:** year: 2013; month: 2; day: 12; eventRemarks: A.P.M. Santos & D.M. Takiya col.; **Record Level:** type: PhysicalObject; institutionCode: DZRJ; basisOfRecord: PreservedSpecimen

#### Distribution

Colombia, Trinidad & Tobago, Panama, Guyana, Suriname, Brazil, Ecuador, Peru, Bolivia, Paraguay, Argentina.

**Distribution in Brazil:** AP, PA, AM, PI!, MT, RO, BA, MG, MS, SP, RJ.

### Potamobates spiculus

Polhemus & Polhemus, 1983

#### Materials

- **Type status:** Other material. **Occurrence:** individualCount: 1; sex: 1 apterous female; **Taxon:** genus: Potamobates; specificEpithet: spiculus; **Location:** continent: South America; country: Peru; stateProvince: Cusco; locality: 3 rd km E Quincemil; decimalLatitude: -13.2208; decimalLongitude: -70.7311; **Identification:** identifiedBy: F.F.F. Moreira; **Event:** year: 2012; month: 8; day: 20; eventRemarks: D. Takiya & A.P.M. Santos col.; **Record Level:** type: PhysicalObject; institutionCode: DZRJ; basisOfRecord: PreservedSpecimen

#### Distribution

Peru.

**Distribution in Peru:** Cusco.

### Rheumatobates bonariensis

(Berg, 1898)

#### Materials

- **Type status:** Other material. **Occurrence:** individualCount: 1; sex: 1 macropterous male; **Taxon:** genus: Rheumatobates; specificEpithet: bonariensis; **Location:** continent: South America; country: Brazil; stateProvince: Mato Grosso; municipality: Barra do Garças; locality: Papagaio, 4ª ordem, superifície 20; **Identification:** identifiedBy: F.F.F. Moreira; **Event:** year: 2007; month: 11; day: 6; eventRemarks: K. Dias-Silva col.; **Record Level:** type: PhysicalObject; institutionCode: DZRJ; basisOfRecord: PreservedSpecimen

#### Distribution

Brazil, Peru, Bolivia, Paraguay, Argentina, Uruguay.

**Distribution in Brazil:** MT!, SP, SC, RS.

### Rheumatobates minutus flavidus

Drake & Harris, 1942

#### Materials

- **Type status:** Other material. **Occurrence:** individualCount: 4; sex: 4 apterous males; **Taxon:** genus: Rheumatobates; specificEpithet: minutus; infraspecificEpithet: flavidus; **Location:** continent: South America; country: Brazil; stateProvince: São Paulo; municipality: Ubatuba; locality: Parque Estadual da Serra do Mar, Núcleo Picinguaba, Riacho Paciência (caminho ao lado do camping); **Identification:** identifiedBy: F.F.F. Moreira; **Event:** year: 2007; month: 10; day: 26; eventRemarks: V.P. Alecrim col.; **Record Level:** type: PhysicalObject; institutionCode: DZRJ; basisOfRecord: PreservedSpecimen

#### Distribution

Costa Rica, Panama, Brazil, Peru, Bolivia, Argentina.

**Distribution in Brazil:** PA, AM, RO, MG, SP!.

### Hydrometra fruhstorferi

Hungerford & Evans, 1934

#### Materials

- **Type status:** Other material. **Occurrence:** individualCount: 1; sex: 1 apterous male; **Taxon:** genus: Hydrometra; specificEpithet: fruhstorferi; **Location:** continent: South America; country: Brazil; stateProvince: Paraná; municipality: Céu Azul; locality: Parque Nacional do Iguaçu, Rio Manoel Gomes; decimalLatitude: -25.1586; decimalLongitude: -53.8331; **Identification:** identifiedBy: F.F.F. Moreira; **Event:** year: 2012; month: 9; day: 7; eventRemarks: B.H.L. Sampaio col.; **Record Level:** type: PhysicalObject; institutionCode: DZRJ; basisOfRecord: PreservedSpecimen

#### Distribution

Brazil, Bolivia, Paraguay, Argentina.

**Distribution in Brazil:** ES, RJ, PR!, SC.

### Mesovelia amoena

Uhler, 1894

#### Materials

- **Type status:** Other material. **Occurrence:** individualCount: 1; sex: 1 apterous female; **Taxon:** genus: Mesovelia; specificEpithet: amoena; **Location:** continent: South America; country: Brazil; stateProvince: Amazonas; municipality: Rio Preto da Eva; locality: Bacia do Rio Urubu, Igarapé de primeira ordem; decimalLatitude: -2.43056; decimalLongitude: -59.62250; **Identification:** identifiedBy: F.F.F. Moreira; **Event:** eventRemarks: J.L. Nessimian col.; **Record Level:** type: PhysicalObject; institutionCode: DZRJ; basisOfRecord: PreservedSpecimen
- **Type status:** Other material. **Occurrence:** individualCount: 1; sex: 1 apterous female; **Taxon:** genus: Mesovelia; specificEpithet: amoena; **Location:** continent: South America; country: Brazil; stateProvince: Bahia; municipality: São Desidério; locality: Gruta do Juraci; decimalLatitude: -12.4102; decimalLongitude: -44.8505; **Identification:** identifiedBy: F.F.F. Moreira; **Event:** year: 2011; month: 11; day: 3; eventRemarks: M.E. Bichuette, D.R. Pedroso, F.F. Pellegatti & T.L.C. Scatolini: col.; **Record Level:** type: PhysicalObject; institutionCode: LES; basisOfRecord: PreservedSpecimen

#### Distribution

Canada, United States, Mexico, Cuba, Dominican Republic, Puerto Rico, Jamaica, Belize, St. Eustatius, Martinique, St. Vincent & Grenadines, Colombia, Curaçao, Bonaire, Grenada, Costa Rica, Trinidad & Tobago, Panama, Brazil, Ecuador, Hawaiian Islands.

**Distribution in Brazil:** PA, AM, CE, MT, RO, BA!, MG, ES, SP, RJ.

### Mesovelia mulsanti

White, 1879

#### Materials

- **Type status:** Other material. **Occurrence:** individualCount: 1; sex: 1 apterous female; **Taxon:** genus: Mesovelia; specificEpithet: mulsanti; **Location:** continent: South America; country: Brazil; stateProvince: Pará; municipality: Canaã dos Carajás; locality: Floresta Nacional de Carajás, Serra Sul, S11B-A; **Identification:** identifiedBy: F.F.F. Moreira; **Event:** year: 2006; month: 10; day: 15; eventRemarks: L.L. Dumas col.; **Record Level:** type: PhysicalObject; institutionCode: DZRJ; basisOfRecord: PreservedSpecimen
- **Type status:** Other material. **Occurrence:** individualCount: 1; sex: 1 apterous female; **Taxon:** genus: Mesovelia; specificEpithet: mulsanti; **Location:** continent: South America; country: Brazil; stateProvince: Pará; municipality: Canaã dos Carajás; locality: Floresta Nacional de Carajás, Serra Sul, S11D-B; **Identification:** identifiedBy: F.F.F. Moreira; **Event:** year: 2011; month: 4; **Record Level:** type: PhysicalObject; institutionCode: DZRJ; basisOfRecord: PreservedSpecimen

#### Distribution

Canada, United States, Mexico, Cuba, Dominican Republic, Puerto Rico, U.S. Virgin Islands, Jamaica, Belize, Guatemala, Honduras, St. Martin, St. Kitts & Nevis, Antigua & Barbuda, Guadeloupe, Nicaragua, Dominica, St. Lucia, St. Vincent & Grenadines, Barbados, Aruba, Colombia, Curaçao, Klein Curaçao, Bonaire, Grenada, Venezuela, Costa Rica, Trinidad & Tobago, Panama, Guyana, Brazil, Peru, Bolivia, Paraguay, Argentina, Hawaiian Islands.

**Distribution in Brazil:** AP, PA, AM, CE, PE, MT, RO, BA, GO, MG, MS, SP, RJ, PR, SC, RS.

### Euvelia lata

Polhemus & Polhemus, 1984

#### Materials

- **Type status:** Other material. **Occurrence:** individualCount: 2; sex: 2 apterous males; **Taxon:** genus: Euvelia; specificEpithet: lata; **Location:** continent: South America; country: Brazil; stateProvince: Amazonas; municipality: Rio Preto da Eva; locality: Bacia do Rio Preto da Eva, Igarapé de segunda ordem; decimalLatitude: -2.64444; decimalLongitude: -59.83528; **Identification:** identifiedBy: F.F.F. Moreira; **Event:** year: 2004; month: 4; day: 22; eventRemarks: J.L. Nessimian col.; **Record Level:** type: PhysicalObject; institutionCode: DZRJ; basisOfRecord: PreservedSpecimen

#### Distribution

Brazil.

**Distribution in Brazil:** PA, AM, MT.

### Microvelia ayacuchana

Drake & Maldonado-Capriles, 1952

#### Materials

- **Type status:** Other material. **Occurrence:** individualCount: 2; sex: 1 apterous male, 1 apterous female; **Taxon:** genus: Microvelia; specificEpithet: ayacuchana; **Location:** continent: South America; country: Brazil; stateProvince: Piauí; municipality: Piracuruca; locality: Parque Nacional de Sete Cidades, Cachoeira do Riachão; verbatimElevation: 171 m; decimalLatitude: -4.1078; decimalLongitude: -41.6736; **Identification:** identifiedBy: F.F.F. Moreira; **Event:** year: 2013; month: 2; day: 9; eventRemarks: A.P.M. Santos & D.M. Takiya col.; **Record Level:** type: PhysicalObject; institutionCode: DZRJ; basisOfRecord: PreservedSpecimen

#### Distribution

Venezuela, Guyana, Suriname, Brazil.

**Distribution in Brazil:** PA, PI!, ES.

### Microvelia longipes

Uhler, 1893

#### Materials

- **Type status:** Other material. **Occurrence:** individualCount: 1; sex: 1 macropterous female; **Taxon:** genus: Microvelia; specificEpithet: longipes; **Location:** continent: South America; country: Brazil; stateProvince: Amazonas; municipality: Rio Preto da Eva; locality: Bacia do Rio Preto da Eva, Igarapé de primeira ordem, armadilha U.V.; decimalLatitude: -2.76306; decimalLongitude: -59.70361; **Identification:** identifiedBy: F.F.F. Moreira; **Event:** year: 2004; month: 4; day: 24; eventRemarks: J.L. Nessimian col.; **Record Level:** type: PhysicalObject; institutionCode: DZRJ; basisOfRecord: PreservedSpecimen
- **Type status:** Other material. **Occurrence:** individualCount: 9; sex: 4 macropterous males, 5 macropterous females; **Taxon:** genus: Microvelia; specificEpithet: longipes; **Location:** continent: South America; country: Brazil; stateProvince: Amazonas; municipality: Manaus; locality: Bacia do Rio Urubu, Igarapé de primeira ordem, armadilha U.V.; decimalLatitude: -2.39889; decimalLongitude: -60.06250; **Identification:** identifiedBy: F.F.F. Moreira; **Event:** year: 2004; month: 5; day: 24; eventRemarks: J.L. Nessimian & L. Fidelis col.; **Record Level:** type: PhysicalObject; institutionCode: DZRJ; basisOfRecord: PreservedSpecimen

#### Distribution

Cuba, Dominican Republic, Puerto Rico, U.S. Virgin Islands, Jamaica, St. Martin, St. Barthélemy, St. Eustatius, St. Kitts & Nevis, Aruba, Colombia, Barbados, Curaçao, Bonaire, Grenada, Venezuela, Trinidad & Tobago, Guyana, Brazil, Ecuador, Peru, Bolivia, Paraguay, Argentina.

**Distribution in Brazil:** RR, AM, BA, MG, MS, ES, SP, RJ, SC.

### Microvelia mimula

White, 1879

#### Materials

- **Type status:** Other material. **Occurrence:** individualCount: 1; sex: 1 macropterous male; **Taxon:** genus: Microvelia; specificEpithet: mimula; **Location:** continent: South America; country: Brazil; stateProvince: Amazonas; municipality: Rio Preto da Eva; locality: Bacia do Rio Urubu, Igarapé Jangada, armadilha U.V.; decimalLatitude: -2.52361; decimalLongitude: -59.66167; **Identification:** identifiedBy: F.F.F. Moreira; **Event:** year: 2004; month: 5; day: 28; eventRemarks: J.L. Nessimian col.; **Record Level:** type: PhysicalObject; institutionCode: DZRJ; basisOfRecord: PreservedSpecimen

#### Distribution

Cuba, Puerto Rico, St. Vincent & Grenadines, Barbados, Grenada, Venezuela, Costa Rica, Trinidad & Tobago, Panama, Brazil, Ecuador, Peru, Paraguay, Argentina, Uruguay.

**Distribution in Brazil:** PA, AM, CE, MT, MG, MS, ES, SP, RJ, SC.

### Microvelia pulchella

Westwood, 1834

#### Materials

- **Type status:** Other material. **Occurrence:** individualCount: 11; sex: 4 apterous males, 6 apterous females, 1 macropterous female; **Taxon:** genus: Microvelia; specificEpithet: pulchella; **Location:** continent: South America; country: Brazil; stateProvince: Pará; municipality: Canaã dos Carajás; locality: Floresta Nacional dos Carajás, Serra Sul; **Identification:** identifiedBy: F.F.F. Moreira; **Event:** year: 2012; month: 11; eventRemarks: Lab. de Limnologia col.; **Record Level:** type: PhysicalObject; institutionCode: DZRJ; basisOfRecord: PreservedSpecimen
- **Type status:** Other material. **Occurrence:** individualCount: 6; sex: 1 macropterous male, 5 macropterous females; **Taxon:** genus: Microvelia; specificEpithet: pulchella; **Location:** continent: South America; country: Brazil; stateProvince: Amazonas; municipality: Rio Preto da Eva; locality: Bacia do Rio Preto da Eva, Igarapé de primeira ordem, armadilha U.V.; decimalLatitude: -2.76306; decimalLongitude: -59.70361; **Identification:** identifiedBy: F.F.F. Moreira; **Event:** year: 2004; month: 4; day: 24; eventRemarks: J.L. Nessimian col.; **Record Level:** type: PhysicalObject; institutionCode: DZRJ; basisOfRecord: PreservedSpecimen
- **Type status:** Other material. **Occurrence:** individualCount: 1; sex: 1 macropterous male; **Taxon:** genus: Microvelia; specificEpithet: pulchella; **Location:** continent: South America; country: Brazil; stateProvince: Amazonas; municipality: Rio Preto da Eva; locality: Bacia do Rio Preto da Eva, Igarapé de terceira ordem, armadilha U.V.; decimalLatitude: -2.72639; decimalLongitude: -59.88083; **Identification:** identifiedBy: F.F.F. Moreira; **Event:** year: 2004; month: 4; day: 23; eventRemarks: J.L. Nessimian col.; **Record Level:** type: PhysicalObject; institutionCode: DZRJ; basisOfRecord: PreservedSpecimen
- **Type status:** Other material. **Occurrence:** individualCount: 2; sex: 2 macropterous males; **Taxon:** genus: Microvelia; specificEpithet: pulchella; **Location:** continent: South America; country: Brazil; stateProvince: Amazonas; municipality: Rio Preto da Eva; locality: Bacia do Rio Urubu, Igarapé Buriti, armadilha U.V.; decimalLatitude: -2.31028; decimalLongitude: -59.86639; **Identification:** identifiedBy: F.F.F. Moreira; **Event:** year: 2004; month: 5; day: 25; eventRemarks: J.L. Nessimian col.; **Record Level:** type: PhysicalObject; institutionCode: DZRJ; basisOfRecord: PreservedSpecimen
- **Type status:** Other material. **Occurrence:** individualCount: 2; sex: 2 macropterous males; **Taxon:** genus: Microvelia; specificEpithet: pulchella; **Location:** continent: South America; country: Brazil; stateProvince: Amazonas; municipality: Rio Preto da Eva; locality: Bacia do Rio Urubu, Igarapé Jangada, armadilha U.V.; decimalLatitude: -2.52361; decimalLongitude: -59.66167; **Identification:** identifiedBy: F.F.F. Moreira; **Event:** year: 2004; month: 5; day: 28; eventRemarks: J.L. Nessimian col.; **Record Level:** type: PhysicalObject; institutionCode: DZRJ; basisOfRecord: PreservedSpecimen
- **Type status:** Other material. **Occurrence:** individualCount: 4; sex: 2 macropterous males, 2 macropterous females; **Taxon:** genus: Microvelia; specificEpithet: pulchella; **Location:** continent: South America; country: Brazil; stateProvince: Amazonas; municipality: Manaus; locality: Bacia do Rio Urubu, Igarapé de primeira ordem, armadilha U.V.; decimalLatitude: -2.39889; decimalLongitude: -60.06250; **Identification:** identifiedBy: F.F.F. Moreira; **Event:** year: 2004; month: 5; day: 24; eventRemarks: J.L. Nessimian & L. Fidelis col.; **Record Level:** type: PhysicalObject; institutionCode: DZRJ; basisOfRecord: PreservedSpecimen
- **Type status:** Other material. **Occurrence:** individualCount: 2; sex: 1 macropterous male, 1 macropterous female; **Taxon:** genus: Microvelia; specificEpithet: pulchella; **Location:** continent: South America; country: Brazil; stateProvince: Amazonas; municipality: Manaus; locality: Bacia do Rio Cuieiras, Igarapé Piranga, armadilha U.V.; decimalLatitude: -2.63907; decimalLongitude: -60.28022; **Identification:** identifiedBy: F.F.F. Moreira; **Event:** year: 2004; month: 8; day: 21; eventRemarks: J.L. Nessimian & L. Fidelis col.; **Record Level:** type: PhysicalObject; institutionCode: DZRJ; basisOfRecord: PreservedSpecimen
- **Type status:** Other material. **Occurrence:** individualCount: 2; sex: 2 macropterous males; **Taxon:** genus: Microvelia; specificEpithet: pulchella; **Location:** continent: South America; country: Brazil; stateProvince: Amazonas; municipality: Manaus; locality: Bacia do Rio Cuieiras, igarapé tributário do Igarapé Lobisomem, armadilha U.V.; decimalLatitude: -2.56288; decimalLongitude: -60.31760; **Identification:** identifiedBy: F.F.F. Moreira; **Event:** year: 2004; month: 8; day: 22; eventRemarks: J.L. Nessimian & L. Fidelis col.; **Record Level:** type: PhysicalObject; institutionCode: DZRJ; basisOfRecord: PreservedSpecimen
- **Type status:** Other material. **Occurrence:** individualCount: 7; sex: 2 apterous males, 3 macropterous males, 2 macropterous females; **Taxon:** genus: Microvelia; specificEpithet: pulchella; **Location:** continent: South America; country: Brazil; stateProvince: Piauí; municipality: Piracuruca; locality: Parque Nacional de Sete Cidades, Rio da Bandeira; verbatimElevation: 189 m; decimalLatitude: -4.0964; decimalLongitude: -41.6833; **Identification:** identifiedBy: F.F.F. Moreira; **Event:** year: 2013; month: 2; day: 8; eventRemarks: A.P.M. Santos & D.M. Takiya col.; **Record Level:** type: PhysicalObject; institutionCode: DZRJ; basisOfRecord: PreservedSpecimen

#### Distribution

Canada, United States, Mexico, Bahamas, Cuba, Dominican Republic, Puerto Rico, U.S. Virgin Islands, Guatemala, Cayman Islands, Jamaica, Anguilla, St. Martin, Saba, St. Kitts & Nevis, Guadeloupe, Martinique, Aruba, Colombia, St. Vincent & Grenadines, Barbados, Curaçao, Klein Curaçao, Bonaire, Klein Bonaire, Grenada, Venezuela, Costa Rica, Trinidad & Tobago, Panama, Brazil, Ecuador, Peru, Argentina.

**Distribution in Brazil:** PA, AM, MA, PI!, PE, BA, AL, MG, MS, ES, SP, RJ, SC.

### Microvelia summersi

Drake & Harris, 1928

#### Materials

- **Type status:** Other material. **Occurrence:** individualCount: 1; sex: 1 macropterous male; **Taxon:** genus: Microvelia; specificEpithet: summersi; **Location:** continent: South America; country: Brazil; stateProvince: Amazonas; municipality: Manaus; locality: Bacia do Rio Preto da Eva, igarapé de terceira ordem, armadilha U.V.; decimalLatitude: -2.72639; decimalLongitude: -59.88083; **Identification:** identifiedBy: F.F.F. Moreira; **Event:** year: 2004; month: 4; day: 23; eventRemarks: J.L. Nessimian col.; **Record Level:** type: PhysicalObject; institutionCode: DZRJ; basisOfRecord: PreservedSpecimen
- **Type status:** Other material. **Occurrence:** individualCount: 1; sex: 1 macropterous male; **Taxon:** genus: Microvelia; specificEpithet: summersi; **Location:** continent: South America; country: Brazil; stateProvince: Amazonas; municipality: Manaus; locality: Bacia do Rio Cuieiras, armadilha U.V.; decimalLatitude: -2.70696; decimalLongitude: -60.3745; **Identification:** identifiedBy: F.F.F. Moreira; **Event:** year: 2004; month: 8; day: 19; eventRemarks: J.L. Nessimian & L. Fidelis col.; **Record Level:** type: PhysicalObject; institutionCode: DZRJ; basisOfRecord: PreservedSpecimen
- **Type status:** Other material. **Occurrence:** individualCount: 2; sex: 2 macropterous males; **Taxon:** genus: Microvelia; specificEpithet: summersi; **Location:** continent: South America; country: Brazil; stateProvince: Amazonas; municipality: Manaus; locality: Bacia do Rio Cuieiras, igarapé de segunda ordem, armadilha U.V.; decimalLatitude: -2.53612; decimalLongitude: -60.31720; **Identification:** identifiedBy: F.F.F. Moreira; **Event:** year: 2004; month: 8; day: 23; eventRemarks: J.L. Nessimian & L. Fidelis col.; **Record Level:** type: PhysicalObject; institutionCode: DZRJ; basisOfRecord: PreservedSpecimen
- **Type status:** Other material. **Occurrence:** individualCount: 60; sex: 12 macropterous males, 48 macropterous females; **Taxon:** genus: Microvelia; specificEpithet: summersi; **Location:** continent: South America; country: Brazil; stateProvince: Amazonas; municipality: Manaus; locality: Bacia do Rio Cuieiras, Igarapé Arumã, armadilha U.V.; decimalLatitude: -2.51532; decimalLongitude: -60.26233; **Identification:** identifiedBy: F.F.F. Moreira; **Event:** year: 2004; month: 8; day: 23; eventRemarks: J.L. Nessimian & L. Fidelis col.; **Record Level:** type: PhysicalObject; institutionCode: DZRJ; basisOfRecord: PreservedSpecimen

#### Distribution

Grenada, Trinidad & Tobago, Panama, Guyana, Brazil.

**Distribution in Brazil:** PA, AM.

### Microvelia ubatuba

Moreira & Barbosa, 2011

#### Materials

- **Type status:** Other material. **Occurrence:** individualCount: 12; sex: 3 macropterous males, 9 macropterous females; **Taxon:** genus: Microvelia; specificEpithet: ubatuba; **Location:** continent: South America; country: Brazil; stateProvince: Amazonas; municipality: Manaus; locality: Bacia do Rio Cuieiras, Igarapé Arumã, armadilha U.V.; decimalLatitude: -2.51532; decimalLongitude: -60.26233; **Identification:** identifiedBy: F.F.F. Moreira; **Event:** year: 2004; month: 8; day: 23; eventRemarks: J.L. Nessimian & L. Fidelis col.; **Record Level:** type: PhysicalObject; institutionCode: DZRJ; basisOfRecord: PreservedSpecimen
- **Type status:** Other material. **Occurrence:** individualCount: 12; sex: 3 macropterous males, 9 macropterous females; **Taxon:** genus: Microvelia; specificEpithet: ubatuba; **Location:** continent: South America; country: Brazil; stateProvince: Amazonas; municipality: Manaus; locality: Bacia do Rio Cuieiras, tributário do Rio Branquinho, armadilha U.V.; decimalLatitude: -2.52350; decimalLongitude: -60.33480; **Identification:** identifiedBy: F.F.F. Moreira; **Event:** year: 2004; month: 8; day: 23; eventRemarks: J.L. Nessimian & L. Fidelis col.; **Record Level:** type: PhysicalObject; institutionCode: DZRJ; basisOfRecord: PreservedSpecimen

#### Distribution

Brazil.

**Distribution in Brazil:** AM, MT, SP.

### Microvelia urucara

#### Materials

- **Type status:** Other material. **Occurrence:** individualCount: 4; sex: 2 apterous males, 1 apterous female, 1 macropterous female; **Taxon:** genus: Microvelia; specificEpithet: urucara; **Location:** continent: South America; country: Brazil; stateProvince: Pará; municipality: Parauapebas; locality: Flona de Carajás, Serra Norte, N4-B; **Identification:** identifiedBy: F.F.F. Moreira; **Event:** year: 2012; month: 11; eventRemarks: Laboratório de Limnologia col.; **Record Level:** type: PhysicalObject; institutionCode: DZRJ; basisOfRecord: PreservedSpecimen
- **Type status:** Other material. **Occurrence:** individualCount: 2; sex: 1 macropterous male, 1 macropterous female; **Taxon:** genus: Microvelia; specificEpithet: urucara; **Location:** continent: South America; country: Brazil; stateProvince: Amazonas; municipality: Manaus; locality: Bacia do Rio Cuieiras, Igarapé Arumã, armadilha U.V.; decimalLatitude: -2.51532; decimalLongitude: -60.26233; **Identification:** identifiedBy: F.F.F. Moreira; **Event:** year: 2004; month: 8; day: 23; eventRemarks: J.L. Nessimian & L. Fidelis col.; **Record Level:** type: PhysicalObject; institutionCode: DZRJ; basisOfRecord: PreservedSpecimen

#### Distribution

Brazil.

**Distribution in Brazil:** PA, AM.

### Microvelia venustatis

Drake & Harris, 1933

#### Materials

- **Type status:** Other material. **Occurrence:** individualCount: 2; sex: 2 macropterous females; **Taxon:** genus: Microvelia; specificEpithet: venustatis; **Location:** continent: South America; country: Brazil; stateProvince: Amazonas; municipality: Rio Preto da Eva; locality: Bacia do Rio Preto da Eva, Igarapé de primeira ordem, armadilha U.V.; decimalLatitude: -2.76306; decimalLongitude: -59.70361; **Identification:** identifiedBy: F.F.F. Moreira; **Event:** year: 2004; month: 4; day: 24; eventRemarks: J.L. Nessimian col.; **Record Level:** type: PhysicalObject; institutionCode: DZRJ; basisOfRecord: PreservedSpecimen
- **Type status:** Other material. **Occurrence:** individualCount: 11; sex: 5 macropterous males, 6 macropterous females; **Taxon:** genus: Microvelia; specificEpithet: venustatis; **Location:** continent: South America; country: Brazil; stateProvince: São Paulo; municipality: Ubatuba; locality: Parque Estadual da Serra do Mar, Núcleo Picinguaba, Riacho Paciência (caminho ao lado do camping); **Identification:** identifiedBy: F.F.F. Moreira; **Event:** year: 2007; month: 10; day: 26; eventRemarks: V.P. Alecrim col.; **Record Level:** type: PhysicalObject; institutionCode: DZRJ; basisOfRecord: PreservedSpecimen

#### Distribution

Brazil, Peru, Paraguay, Argentina.

**Distribution in Brazil:** PA, AM, MA, MT, MG, ES, SP, RJ, SC.

### Platyvelia brachialis

#### Materials

- **Type status:** Other material. **Occurrence:** individualCount: 1; sex: 1 macropterous female; **Taxon:** genus: Platyvelia; specificEpithet: brachialis; **Location:** continent: South America; country: Brazil; stateProvince: Piauí; municipality: Piracuruca; locality: Parque Nacional de Sete Cidades, Olho d'água dos milagres; verbatimElevation: 180 m; decimalLatitude: -4.0919; decimalLongitude: -41.6833; **Identification:** identifiedBy: F.F.F. Moreira; **Event:** year: 2013; month: 2; day: 11; eventRemarks: A.P.M. Santos & D.M. Takiya col.; **Record Level:** type: PhysicalObject; institutionCode: DZRJ; basisOfRecord: PreservedSpecimen

#### Distribution

United States, Mexico, Cuba, Dominican Republic, Jamaica, Guatemala, Nicaragua, Grenada, Costa Rica, Trinidad & Tobago, Panama, Suriname, Brazil, Peru, Argentina.

**Distribution in Brazil:** PI!, PE, MT, GO, MG, MS, ES, RJ, SC.

### Rhagovelia accedens

#### Materials

- **Type status:** Other material. **Occurrence:** individualCount: 3; sex: 2 apterous males, 1 apterous female; **Taxon:** genus: Rhagovelia; specificEpithet: accedens; **Location:** continent: South America; country: Brazil; stateProvince: Espírito Santo; municipality: Dores do Rio Preto; locality: Parque Nacional do Caparaó, Pedra Menina, Rio Preto (ponte); **Identification:** identifiedBy: F.F.F. Moreira; **Event:** year: 2013; month: 1; day: 7; eventRemarks: A.L.H. Oliveira col.; **Record Level:** type: PhysicalObject; institutionCode: DZRJ; basisOfRecord: PreservedSpecimen

#### Distribution

Brazil.

**Distribution in Brazil:** MG, ES, SP, RJ.

### Rhagovelia aiuruoca

Moreira & Ribeiro, 2009

#### Materials

- **Type status:** Other material. **Occurrence:** individualCount: 15; sex: 8 apterous males, 7 apterous females; **Taxon:** genus: Rhagovelia; specificEpithet: aiuruoca; **Location:** continent: South America; country: Brazil; stateProvince: Minas Gerais; municipality: Alto Caparaó; locality: Parque Nacional do Caparaó, Rio José Pedro, Cachoeira Bonita; **Identification:** identifiedBy: F.F.F. Moreira; **Event:** year: 2010; month: 10; day: 5; eventRemarks: J.L. Nessimian & L.L. Dumas col.; **Record Level:** type: PhysicalObject; institutionCode: DZRJ; basisOfRecord: PreservedSpecimen

#### Distribution

Brazil

**Distribution in Brazil:** MG, SP, RJ.

### Rhagovelia elegans

Uhler, 1894

#### Materials

- **Type status:** Other material. **Occurrence:** individualCount: 1; sex: 1 apterous female; **Taxon:** genus: Rhagovelia; specificEpithet: elegans; **Location:** continent: South America; country: Brazil; stateProvince: Amazonas; municipality: Rio Preto da Eva; locality: Bacia do Rio Preto da Eva!, igarapé de primeira ordem; decimalLatitude: -2.76306; decimalLongitude: -59.70361; **Identification:** identifiedBy: F.F.F. Moreira; **Event:** year: 2004; month: 4; day: 24; eventRemarks: J.L. Nessimian col.; **Record Level:** type: PhysicalObject; institutionCode: DZRJ; basisOfRecord: PreservedSpecimen
- **Type status:** Other material. **Occurrence:** individualCount: 2; sex: 2 apterous females; **Taxon:** genus: Rhagovelia; specificEpithet: elegans; **Location:** continent: South America; country: Brazil; stateProvince: Amazonas; municipality: Manaus; locality: Bacia do Rio Cuieiras, Polícia Federal, igarapé de primeira ou segunda ordem; decimalLatitude: -2.70534; decimalLongitude: -60.38372; **Identification:** identifiedBy: F.F.F. Moreira; **Event:** year: 2004; month: 8; day: 19; eventRemarks: J.L. Nessimian & L. Fidelis col.; **Record Level:** type: PhysicalObject; institutionCode: DZRJ; basisOfRecord: PreservedSpecimen
- **Type status:** Other material. **Occurrence:** individualCount: 2; sex: 2 apterous females; **Taxon:** genus: Rhagovelia; specificEpithet: elegans; **Location:** continent: South America; country: Brazil; stateProvince: Amazonas; municipality: Manaus; locality: Bacia do Rio Cuieiras, Igarapé de primeira ordem; decimalLatitude: -2.70696; decimalLongitude: -60.37450; **Identification:** identifiedBy: F.F.F. Moreira; **Event:** year: 2004; month: 8; day: 19; eventRemarks: J.L. Nessimian & L. Fidelis col.; **Record Level:** type: PhysicalObject; institutionCode: DZRJ; basisOfRecord: PreservedSpecimen
- **Type status:** Other material. **Occurrence:** individualCount: 1; sex: 1 apterous female; **Taxon:** genus: Rhagovelia; specificEpithet: elegans; **Location:** continent: South America; country: Brazil; stateProvince: Amazonas; municipality: Manaus; locality: Bacia do Rio Cuieiras, Tributário do Igarapé da Cachoeira; decimalLatitude: -2.69593; decimalLongitude: -60.29520; **Identification:** identifiedBy: F.F.F. Moreira; **Event:** year: 2004; month: 8; day: 20; eventRemarks: J.L. Nessimian & L. Fidelis col.; **Record Level:** type: PhysicalObject; institutionCode: DZRJ; basisOfRecord: PreservedSpecimen
- **Type status:** Other material. **Occurrence:** individualCount: 1; sex: 1 macropterous male; **Taxon:** genus: Rhagovelia; specificEpithet: elegans; **Location:** continent: South America; country: Brazil; stateProvince: Amazonas; municipality: Manaus; locality: Bacia do Rio Cuieiras, Igarapé do Paliteiro, Tributário do Rio Branquinho; decimalLatitude: -2.45865; decimalLongitude: -60.34600; **Identification:** identifiedBy: F.F.F. Moreira; **Event:** year: 2004; month: 8; day: 26; eventRemarks: J.L. Nessimian & L. Fidelis col.; **Record Level:** type: PhysicalObject; institutionCode: DZRJ; basisOfRecord: PreservedSpecimen
- **Type status:** Other material. **Occurrence:** individualCount: 2; sex: 1 macropterous male, 1 macropterous female; **Taxon:** genus: Rhagovelia; specificEpithet: elegans; **Location:** continent: South America; country: Brazil; stateProvince: Amazonas; municipality: Manaus; locality: Bacia do Rio Cuieiras, Igarapé da Onça, tributário do Rio Branquinho; decimalLatitude: -2.49308; decimalLongitude: -60.33419; **Identification:** identifiedBy: F.F.F. Moreira; **Event:** year: 2004; month: 8; day: 26; eventRemarks: J.L. Nessimian & L. Fidelis col.; **Record Level:** type: PhysicalObject; institutionCode: DZRJ; basisOfRecord: PreservedSpecimen

#### Distribution

Hispaniola, St. Kitts & Nevis, Dominica, Martinique, St. Lucia, St. Vincent & Grenadines, Colombia, Grenada, Venezuela, Costa Rica, Trinidad & Tobago, Panama, Brazil, Ecuador.

**Distribution in Brazil:** AP, PA, AM, MT, SE, ES, RJ.

### Rhagovelia fontanalis

#### Materials

- **Type status:** Other material. **Occurrence:** individualCount: 21; sex: 9 apterous males, 12 apterous females; **Taxon:** genus: Rhagovelia; specificEpithet: fontanalis; **Location:** continent: South America; country: Peru; stateProvince: Madre de Dios; locality: 12 rd km E Mazuko, pte. Amanapu; decimalLatitude: -13.0442; decimalLongitude: -70.3128; **Identification:** identifiedBy: F.F.F. Moreira; **Event:** year: 2012; month: 8; day: 18; eventRemarks: D. Takiya & A.P.M. Santos col.; **Record Level:** type: PhysicalObject; institutionCode: DZRJ; basisOfRecord: PreservedSpecimen

#### Distribution

Peru.

**Distribution in Peru:** Ucayali, Huánuco, *Madre de Dios*!, Junin, Ayacucho.

### Rhagovelia itatiaiana

Drake, 1953

#### Materials

- **Type status:** Other material. **Occurrence:** individualCount: 12; sex: 6 apterous males, 6 apterous females; **Taxon:** genus: Rhagovelia; specificEpithet: itatiaiana; **Location:** continent: South America; country: Brazil; stateProvince: Rio de Janeiro; municipality: Rio de Janeiro; locality: Jacarepaguá, Taquara, Estrada Pau da Fome, Parque Estadual da Pedra Branca, Rio Manuel Justino; decimalLatitude: -22.9365; decimalLongitude: -43.4447; **Identification:** identifiedBy: I.R.S. Cordeiro; **Event:** year: 2012; month: 11; day: 19; eventRemarks: I.R.S. Cordeiro & F.A.C. Silva col.; **Record Level:** type: PhysicalObject; institutionCode: DZRJ; basisOfRecord: PreservedSpecimen
- **Type status:** Other material. **Occurrence:** individualCount: 14; sex: 8 apterous males, 6 apterous females; **Taxon:** genus: Rhagovelia; specificEpithet: itatiaiana; **Location:** continent: South America; country: Brazil; stateProvince: Rio de Janeiro; municipality: Rio de Janeiro; locality: Jacarepaguá, Taquara, Estrada Pau da Fome, Parque Estadual da Pedra Branca, Rio Manuel Justino; decimalLatitude: -22.9365; decimalLongitude: -43.4447; **Identification:** identifiedBy: I.R.S. Cordeiro; **Event:** year: 2013; month: 5; day: 26; eventRemarks: I.R.S. Cordeiro, F.A.C. Silva, F.F. Silva, L. Chaves & A. Ferreira col.; **Record Level:** type: PhysicalObject; institutionCode: DZRJ; basisOfRecord: PreservedSpecimen
- **Type status:** Other material. **Occurrence:** individualCount: 5; sex: 5 apterous males; **Taxon:** genus: Rhagovelia; specificEpithet: itatiaiana; **Location:** continent: South America; country: Brazil; stateProvince: Rio de Janeiro; municipality: Rio de Janeiro; locality: Jacarepaguá, Taquara, Estrada Pau da Fome, Parque Estadual da Pedra Branca, Rio Manuel Justino; decimalLatitude: -22.9365; decimalLongitude: -43.4447; **Identification:** identifiedBy: I.R.S. Cordeiro; **Event:** year: 2013; month: 9; day: 7; eventRemarks: I.R.S. Cordeiro, F.A.C. Silva, F.F. Silva, L. Chaves & A. Ferreira col.; **Record Level:** type: PhysicalObject; institutionCode: DZRJ; basisOfRecord: PreservedSpecimen
- **Type status:** Other material. **Occurrence:** individualCount: 19; sex: 7 apterous males, 12 apterous females; **Taxon:** genus: Rhagovelia; specificEpithet: itatiaiana; **Location:** continent: South America; country: Brazil; stateProvince: Rio de Janeiro; municipality: Rio de Janeiro; locality: Jacarepaguá, Taquara, Estrada Pau da Fome, Parque Estadual da Pedra Branca, Rio Figueira; decimalLatitude: -22.9327; decimalLongitude: -43.4380; **Identification:** identifiedBy: I.R.S. Cordeiro; **Event:** year: 2012; month: 10; day: 15; eventRemarks: I.R.S. Cordeiro & F.A.C. Silva col.; **Record Level:** type: PhysicalObject; institutionCode: DZRJ; basisOfRecord: PreservedSpecimen
- **Type status:** Other material. **Occurrence:** individualCount: 18; sex: 7 apterous males, 11 apterouos females; **Taxon:** genus: Rhagovelia; specificEpithet: itatiaiana; **Location:** continent: South America; country: Brazil; stateProvince: Rio de Janeiro; municipality: Rio de Janeiro; locality: Jacarepaguá, Taquara, Estrada Pau da Fome, Parque Estadual da Pedra Branca, Rio Figueira; decimalLatitude: -22.9327; decimalLongitude: -43.4380; **Identification:** identifiedBy: I.R.S. Cordeiro; **Event:** year: 2013; month: 6; day: 8; eventRemarks: I.R.S. Cordeiro, F.A.C. Silva, F.F. Silva, L. Chaves & A. Ferreira col.; **Record Level:** type: PhysicalObject; institutionCode: DZRJ; basisOfRecord: PreservedSpecimen
- **Type status:** Other material. **Occurrence:** individualCount: 35; sex: 26 apterous males, 9 apterous females; **Taxon:** genus: Rhagovelia; specificEpithet: itatiaiana; **Location:** continent: South America; country: Brazil; stateProvince: Rio de Janeiro; municipality: Rio de Janeiro; locality: Jacarepaguá, Taquara, Estrada Pau da Fome, Parque Estadual da Pedra Branca, Rio Figueira; decimalLatitude: -22.9327; decimalLongitude: -43.4380; **Identification:** identifiedBy: I.R.S. Cordeiro; **Event:** year: 2013; month: 8; day: 19; eventRemarks: I.R.S. Cordeiro, F.A.C. Silva, F.F. Silva, L. Chaves & A. Ferreira col.; **Record Level:** type: PhysicalObject; institutionCode: DZRJ; basisOfRecord: PreservedSpecimen
- **Type status:** Other material. **Occurrence:** individualCount: 37; sex: 22 apterous males, 15 apterous females; **Taxon:** genus: Rhagovelia; specificEpithet: itatiaiana; **Location:** continent: South America; country: Brazil; stateProvince: Rio de Janeiro; municipality: Rio de Janeiro; locality: Jacarepaguá, Taquara, Estrada Pau da Fome, Parque Estadual da Pedra Branca, Rio do Quilombo; decimalLatitude: -22.9323; decimalLongitude: -43.4358; **Identification:** identifiedBy: I.R.S. Cordeiro; **Event:** year: 2013; month: 6; day: 16; eventRemarks: I.R.S. Cordeiro, F.A.C. Silva, F.F. Silva, L. Chaves & A. Ferreira col.; **Record Level:** type: PhysicalObject; institutionCode: DZRJ; basisOfRecord: PreservedSpecimen
- **Type status:** Other material. **Occurrence:** individualCount: 5; sex: 5 apterous males; **Taxon:** genus: Rhagovelia; specificEpithet: itatiaiana; **Location:** continent: South America; country: Brazil; stateProvince: Rio de Janeiro; municipality: Rio de Janeiro; locality: Jacarepaguá, Taquara, Estrada Pau da Fome, Parque Estadual da Pedra Branca, Rio do Quilombo; decimalLatitude: -22.9323; decimalLongitude: -43.4358; **Identification:** identifiedBy: I.R.S. Cordeiro; **Event:** year: 2013; month: 8; day: 24; eventRemarks: I.R.S. Cordeiro, F.A.C. Silva, F.F. Silva, L. Chaves & A. Ferreira col.; **Record Level:** type: PhysicalObject; institutionCode: DZRJ; basisOfRecord: PreservedSpecimen
- **Type status:** Other material. **Occurrence:** individualCount: 9; sex: 3 apterous males, 6 apterous females; **Taxon:** genus: Rhagovelia; specificEpithet: itatiaiana; **Location:** continent: South America; country: Brazil; stateProvince: Rio de Janeiro; municipality: Rio de Janeiro; locality: Jacarepaguá, Taquara, Estrada Pau da Fome, Parque Estadual da Pedra Branca, Rio Grande; decimalLatitude: -22.9320; decimalLongitude: -43.4452; **Identification:** identifiedBy: I.R.S. Cordeiro; **Event:** year: 2012; month: 11; day: 19; eventRemarks: I.R.S. Cordeiro & F.A.C. Silva col.; **Record Level:** type: PhysicalObject; institutionCode: DZRJ; basisOfRecord: PreservedSpecimen
- **Type status:** Other material. **Occurrence:** individualCount: 2; sex: 1 apterous male, 1 apterous female; **Taxon:** genus: Rhagovelia; specificEpithet: itatiaiana; **Location:** continent: South America; country: Brazil; stateProvince: Rio de Janeiro; municipality: Rio de Janeiro; locality: Jacarepaguá, Taquara, Estrada Pau da Fome, Parque Estadual da Pedra Branca, Rio Grande; decimalLatitude: -22.9320; decimalLongitude: -43.4452; **Identification:** identifiedBy: I.R.S. Cordeiro; **Event:** year: 2013; month: 2; day: 23; eventRemarks: I.R.S. Cordeiro, F.A.C. Silva, F.F. Silva, L. Chaves & A. Ferreira col.; **Record Level:** type: PhysicalObject; institutionCode: DZRJ; basisOfRecord: PreservedSpecimen
- **Type status:** Other material. **Occurrence:** individualCount: 9; sex: 7 apterous males, 2 apterous females; **Taxon:** genus: Rhagovelia; specificEpithet: itatiaiana; **Location:** continent: South America; country: Brazil; stateProvince: Rio de Janeiro; municipality: Rio de Janeiro; locality: Jacarepaguá, Taquara, Estrada Pau da Fome, Parque Estadual da Pedra Branca, Rio Grande; decimalLatitude: -22.9320; decimalLongitude: -43.4452; **Identification:** identifiedBy: I.R.S. Cordeiro; **Event:** year: 2013; month: 5; day: 26; eventRemarks: I.R.S. Cordeiro, F.A.C. Silva, F.F. Silva, L. Chaves & A. Ferreira col.; **Record Level:** type: PhysicalObject; institutionCode: DZRJ; basisOfRecord: PreservedSpecimen
- **Type status:** Other material. **Occurrence:** individualCount: 2; sex: 1 apterous male, 1 apterous female; **Taxon:** genus: Rhagovelia; specificEpithet: itatiaiana; **Location:** continent: South America; country: Brazil; stateProvince: Rio de Janeiro; municipality: Rio de Janeiro; locality: Jacarepaguá, Taquara, Estrada Pau da Fome, Parque Estadual da Pedra Branca, Rio Grande; decimalLatitude: -22.9320; decimalLongitude: -43.4452; **Identification:** identifiedBy: I.R.S. Cordeiro; **Event:** year: 2013; month: 9; day: 7; eventRemarks: I.R.S. Cordeiro, F.A.C. Silva, F.F. Silva, L. Chaves & A. Ferreira col.; **Record Level:** type: PhysicalObject; institutionCode: DZRJ; basisOfRecord: PreservedSpecimen
- **Type status:** Other material. **Occurrence:** individualCount: 12; sex: 10 apterous males, 2 apterous females; **Taxon:** genus: Rhagovelia; specificEpithet: itatiaiana; **Location:** continent: South America; country: Brazil; stateProvince: Rio de Janeiro; municipality: Rio de Janeiro; locality: Jacarepaguá, Taquara, Estrada Pau da Fome, Parque Estadual da Pedra Branca, Rio Grande; decimalLatitude: -22.9320; decimalLongitude: -43.4452; **Identification:** identifiedBy: I.R.S. Cordeiro; **Event:** year: 2014; month: 4; day: 19; eventRemarks: I.R.S. Cordeiro, F.A.C. Silva, F.F. Silva, L. Chaves, A. Ferreira, L.S. Barbosa, D.M.C. Santos & L.B. Pena Forte col.; **Record Level:** type: PhysicalObject; institutionCode: DZRJ; basisOfRecord: PreservedSpecimen

#### Distribution

Brazil.

**Distribution in Brazil:** ES, RJ.

### Rhagovelia janeira

Drake, 1953

#### Materials

- **Type status:** Other material. **Occurrence:** individualCount: 17; sex: 14 apterous males, 3 apterous females; **Taxon:** genus: Rhagovelia; specificEpithet: janeira; **Location:** continent: South America; country: Brazil; stateProvince: Paraná; municipality: Céu Azul; locality: Parque Nacional do Iguaçu, Rio Manoel Gomes; decimalLatitude: -25.15860; decimalLongitude: -53.83308; **Identification:** identifiedBy: F.F.F. Moreira; **Event:** year: 2012; month: 9; day: 7; eventRemarks: J.F. Barbosa col.; **Record Level:** type: PhysicalObject; institutionCode: DZRJ; basisOfRecord: PreservedSpecimen

#### Distribution

Brazil, Argentina.

**Distribution in Brazil:** MG, SP, RJ, PR!, SC, RS.

### Rhagovelia lucida

Gould, 1931

#### Materials

- **Type status:** Other material. **Occurrence:** individualCount: 1; sex: 1 apterous female; **Taxon:** genus: Rhagovelia; specificEpithet: lucida; **Location:** continent: South America; country: Brazil; stateProvince: Espírito Santo; municipality: Dores do Rio Preto; locality: Parque Nacional Caparaó, Pedra Menina, Rio Preto (ponte); **Identification:** identifiedBy: F.F.F. Moreira; **Event:** year: 2013; month: 1; day: 7; eventRemarks: A.L.H. Oliveira col.; **Record Level:** type: PhysicalObject; institutionCode: DZRJ; basisOfRecord: PreservedSpecimen
- **Type status:** Other material. **Occurrence:** individualCount: 1; sex: 1 apterous male; **Taxon:** genus: Rhagovelia; specificEpithet: lucida; **Location:** continent: South America; country: Brazil; stateProvince: Paraná; municipality: Céu Azul; locality: Parque Nacional do Iguaçu, Rio Azul (3ª ordem); decimalLatitude: -25.15581; decimalLongitude: -53.79230; **Identification:** identifiedBy: F.F.F. Moreira; **Event:** year: 2012; month: 9; day: 6; eventRemarks: J.F. Barbosa col.; **Record Level:** type: PhysicalObject; institutionCode: DZRJ; basisOfRecord: PreservedSpecimen
- **Type status:** Other material. **Occurrence:** individualCount: 3; sex: 1 apterous male, 2 apterous females; **Taxon:** genus: Rhagovelia; specificEpithet: lucida; **Location:** continent: South America; country: Brazil; stateProvince: Rio de Janeiro; municipality: Rio de Janeiro; locality: Jacarepaguá, Taquara, Estrada Pau da Fome, Parque Estadual da Pedra Branca, Rio Figueira; decimalLatitude: -22.9327; decimalLongitude: -43.4380; **Identification:** identifiedBy: I.R.S. Cordeiro; **Event:** year: 2013; month: 6; day: 8; eventRemarks: I.R.S. Cordeiro & F.A.C. Silva col.; **Record Level:** type: PhysicalObject; institutionCode: DZRJ; basisOfRecord: PreservedSpecimen
- **Type status:** Other material. **Occurrence:** individualCount: 21; sex: 9 apterous males, 12 apterous females; **Taxon:** genus: Rhagovelia; specificEpithet: lucida; **Location:** continent: South America; country: Brazil; stateProvince: Rio de Janeiro; municipality: Rio de Janeiro; locality: Jacarepaguá, Taquara, Estrada Pau da Fome, Parque Estadual da Pedra Branca, Rio Figueira; decimalLatitude: -22.9327; decimalLongitude: -43.4380; **Identification:** identifiedBy: I.R.S. Cordeiro; **Event:** year: 2013; month: 8; day: 19; eventRemarks: I.R.S. Cordeiro, F.A.C. Silva, F.F. Silva, L. Chaves & A. Ferreira col.; **Record Level:** type: PhysicalObject; institutionCode: DZRJ; basisOfRecord: PreservedSpecimen
- **Type status:** Other material. **Occurrence:** individualCount: 8; sex: 3 apterous males, 1 macropterous male, 2 apterous females, 2 macropterous females; **Taxon:** genus: Rhagovelia; specificEpithet: lucida; **Location:** continent: South America; country: Brazil; stateProvince: Rio de Janeiro; municipality: Rio de Janeiro; locality: Jacarepaguá, Taquara, Estrada Pau da Fome, Parque Estadual da Pedra Branca, Rio Grande; decimalLatitude: -22.9320; decimalLongitude: -43.4452; **Identification:** identifiedBy: I.R.S. Cordeiro; **Event:** year: 2012; month: 11; day: 19; eventRemarks: I.R.S. Cordeiro & F.A.C. Silva col.; **Record Level:** type: PhysicalObject; institutionCode: DZRJ; basisOfRecord: PreservedSpecimen
- **Type status:** Other material. **Occurrence:** individualCount: 6; sex: 6 apterous males; **Taxon:** genus: Rhagovelia; specificEpithet: lucida; **Location:** continent: South America; country: Brazil; stateProvince: Rio de Janeiro; municipality: Rio de Janeiro; locality: Jacarepaguá, Taquara, Estrada Pau da Fome, Parque Estadual da Pedra Branca, Rio Grande; decimalLatitude: -22.9320; decimalLongitude: -43.4452; **Identification:** identifiedBy: I.R.S. Cordeiro; **Event:** year: 2013; month: 2; day: 23; eventRemarks: I.R.S. Cordeiro, F.A.C. Silva, F.F. Silva, L. Chaves & A. Ferreira col.; **Record Level:** type: PhysicalObject; institutionCode: DZRJ; basisOfRecord: PreservedSpecimen
- **Type status:** Other material. **Occurrence:** individualCount: 7; sex: 3 macropterous males, 4 apterous females; **Taxon:** genus: Rhagovelia; specificEpithet: lucida; **Location:** continent: South America; country: Brazil; stateProvince: Rio de Janeiro; municipality: Rio de Janeiro; locality: Jacarepaguá, Taquara, Estrada Pau da Fome, Parque Estadual da Pedra Branca, Rio Grande; decimalLatitude: -22.9320; decimalLongitude: -43.4452; **Identification:** identifiedBy: I.R.S. Cordeiro; **Event:** year: 2013; month: 5; day: 26; eventRemarks: I.R.S. Cordeiro, F.A.C. Silva, F.F. Silva, L. Chaves & A. Ferreira col.; **Record Level:** type: PhysicalObject; institutionCode: DZRJ; basisOfRecord: PreservedSpecimen
- **Type status:** Other material. **Occurrence:** individualCount: 78; sex: 36 apterous males, 5 macropterous males, 32 apterous females, 5 macropterous females; **Taxon:** genus: Rhagovelia; specificEpithet: lucida; **Location:** continent: South America; country: Brazil; stateProvince: Rio de Janeiro; municipality: Rio de Janeiro; locality: Jacarepaguá, Taquara, Estrada Pau da Fome, Parque Estadual da Pedra Branca, Rio Grande; decimalLatitude: -22.9320; decimalLongitude: -43.4452; **Identification:** identifiedBy: I.R.S. Cordeiro; **Event:** year: 2013; month: 9; day: 7; eventRemarks: I.R.S. Cordeiro, F.A.C. Silva, F.F. Silva, L. Chaves & A. Ferreira col.; **Record Level:** type: PhysicalObject; institutionCode: DZRJ; basisOfRecord: PreservedSpecimen
- **Type status:** Other material. **Occurrence:** individualCount: 56; sex: 35 apterous males, 21 apterous females; **Taxon:** genus: Rhagovelia; specificEpithet: lucida; **Location:** continent: South America; country: Brazil; stateProvince: Rio de Janeiro; municipality: Rio de Janeiro; locality: Jacarepaguá, Taquara, Estrada Pau da Fome, Parque Estadual da Pedra Branca, Rio Grande; decimalLatitude: -22.9320; decimalLongitude: -43.4452; **Identification:** identifiedBy: I.R.S. Cordeiro; **Event:** year: 2014; month: 4; day: 19; eventRemarks: I.R.S. Cordeiro, F.A.C. Silva, F.F. Silva, L. Chaves, A. Ferreira, L.S. Barbosa, D.M.S. Corrêa & L.B. Pena Forte col.; **Record Level:** type: PhysicalObject; institutionCode: DZRJ; basisOfRecord: PreservedSpecimen

#### Distribution

Brazil, Argentina.

**Distribution in Brazil:** ES!, SP, RJ, PR, SC, RS.

### Rhagovelia macta

Drake & Carvalho, 1955

#### Materials

- **Type status:** Other material. **Occurrence:** individualCount: 4; sex: 4 apterous males; **Taxon:** genus: Rhagovelia; specificEpithet: macta; **Location:** continent: South America; country: Brazil; stateProvince: Rio de Janeiro; municipality: Rio de Janeiro; locality: Jacarepaguá, Taquara, Estrada Pau da Fome, Parque Estadual da Pedra Branca, Rio Manuel Justino; decimalLatitude: -22.9365; decimalLongitude: -43.4447; **Identification:** identifiedBy: I.R.S. Cordeiro; **Event:** year: 2013; month: 9; day: 7; eventRemarks: I.R.S. Cordeiro, F.A.C. Silva, F.F. Silva, L. Chaves & A. Ferreira col.; **Record Level:** type: PhysicalObject; institutionCode: DZRJ; basisOfRecord: PreservedSpecimen
- **Type status:** Other material. **Occurrence:** individualCount: 85; sex: 20 apterous males, 65 apterous females; **Taxon:** genus: Rhagovelia; specificEpithet: macta; **Location:** continent: South America; country: Brazil; stateProvince: Rio de Janeiro; municipality: Rio de Janeiro; locality: Jacarepaguá, Taquara, Estrada Pau da Fome, Parque Estadual da Pedra Branca, Rio Figueira; decimalLatitude: -22.9327; decimalLongitude: -43.4380; **Identification:** identifiedBy: I.R.S. Cordeiro; **Event:** year: 2013; month: 8; day: 19; eventRemarks: I.R.S. Cordeiro, F.A.C. Silva, F.F. Silva, L. Chaves & A. Ferreira col.; **Record Level:** type: PhysicalObject; institutionCode: DZRJ; basisOfRecord: PreservedSpecimen
- **Type status:** Other material. **Occurrence:** individualCount: 12; sex: 12 apterous males; **Taxon:** genus: Rhagovelia; specificEpithet: macta; **Location:** continent: South America; country: Brazil; stateProvince: Rio de Janeiro; municipality: Rio de Janeiro; locality: Jacarepaguá, Taquara, Estrada Pau da Fome, Parque Estadual da Pedra Branca, Rio do Quilombo; decimalLatitude: -22.9323; decimalLongitude: -43.4358; **Identification:** identifiedBy: I.R.S. Cordeiro; **Event:** year: 2013; month: 8; day: 24; eventRemarks: I.R.S. Cordeiro, F.A.C. Silva, F.F. Silva, L. Chaves & A. Ferreira col.; **Record Level:** type: PhysicalObject; institutionCode: DZRJ; basisOfRecord: PreservedSpecimen
- **Type status:** Other material. **Occurrence:** individualCount: 2; sex: 2 apterous males; **Taxon:** genus: Rhagovelia; specificEpithet: macta; **Location:** continent: South America; country: Brazil; stateProvince: Rio de Janeiro; municipality: Rio de Janeiro; locality: Jacarepaguá, Taquara, Estrada Pau da Fome, Parque Estadual da Pedra Branca, Rio Grande; decimalLatitude: -22.9320; decimalLongitude: -43.4452; **Identification:** identifiedBy: I.R.S. Cordeiro; **Event:** year: 2014; month: 4; day: 19; eventRemarks: I.R.S. Cordeiro, F.A.C. Silva, F.F. Silva, L. Chaves, A. Ferreira, L.S. Barbosa, D.M.S. Corrêa & L.B. Pena Forte col.; **Record Level:** type: PhysicalObject; institutionCode: DZRJ; basisOfRecord: PreservedSpecimen

#### Distribution

Brazil.

**Distribution in Brazil:** MG, RJ.

### Rhagovelia traili

#### Materials

- **Type status:** Other material. **Occurrence:** individualCount: 1; sex: 1 apterous male; **Taxon:** genus: Rhagovelia; specificEpithet: traili; **Location:** continent: South America; country: Brazil; stateProvince: Amazonas; municipality: Manaus; locality: Bacia do Rio Cuieiras, Igarapé Piranga; decimalLatitude: -2.63907; decimalLongitude: -60.28022; **Identification:** identifiedBy: F.F.F. Moreira; **Event:** year: 2004; month: 8; day: 21; eventRemarks: J.L. Nessimian & L. Fidelis col.; **Record Level:** type: PhysicalObject; institutionCode: DZRJ; basisOfRecord: PreservedSpecimen

#### Distribution

Venezuela, Suriname, French Guiana, Brazil, Peru.

**Distribution in Brazil:** RR, PA, AM.

### Rhagovelia triangula

Drake, 1953

#### Materials

- **Type status:** Other material. **Occurrence:** individualCount: 5; sex: 4 apterous males, 1 apterous female; **Taxon:** genus: Rhagovelia; specificEpithet: triangula; **Location:** continent: South America; country: Brazil; stateProvince: Minas Gerais; municipality: Araponga; locality: Pico do Boné, cachoeira; **Identification:** identifiedBy: F.F.F. Moreira; **Event:** year: 2011; month: 9; day: 24; eventRemarks: V. Sandoval; **Record Level:** type: PhysicalObject; institutionCode: DZRJ; basisOfRecord: PreservedSpecimen
- **Type status:** Other material. **Occurrence:** individualCount: 1; sex: 1 apterous male; **Taxon:** genus: Rhagovelia; specificEpithet: triangula; **Location:** continent: South America; country: Brazil; stateProvince: Minas Gerais; municipality: Alto Caparaó; locality: Parque Nacional Caparaó, Rio José Pedro, Cachoeira Bonita; **Identification:** identifiedBy: F.F.F. Moreira; **Event:** year: 2010; month: 10; day: 5; eventRemarks: J.L. Nessimian & L.L. Dumas col.; **Record Level:** type: PhysicalObject; institutionCode: DZRJ; basisOfRecord: PreservedSpecimen

#### Distribution

Brazil.

**Distribution in Brazil:** MG, ES, SP, RJ.

### Rhagovelia trianguloides

Nieser & Melo, 1997

#### Materials

- **Type status:** Other material. **Occurrence:** individualCount: 1; sex: 1 apterous male; **Taxon:** genus: Rhagovelia; specificEpithet: trianguloides; **Location:** continent: South America; country: Brazil; stateProvince: Minas Gerais; municipality: Araponga; locality: Pico do Boné, cachoeira; **Identification:** identifiedBy: F.F.F. Moreira; **Event:** year: 2011; month: 9; day: 24; eventRemarks: V. Sandoval; **Record Level:** type: PhysicalObject; institutionCode: DZRJ; basisOfRecord: PreservedSpecimen

#### Distribution

Brazil.

**Distribution in Brazil:** MG, ES, RJ.

### Rhagovelia versuta

Drake & Harris, 1935

#### Materials

- **Type status:** Other material. **Occurrence:** individualCount: 2; sex: 1 macropterous male, 1 macropterous male with broken wings; **Taxon:** genus: Rhagovelia; specificEpithet: versuta; **Location:** continent: South America; country: Peru; stateProvince: Cusco; locality: 20 rd Km W Quincemil, Río Araza tributary; decimalLatitude: -13.33694; decimalLongitude: -70.85417; **Identification:** identifiedBy: F.F.F. Moreira; **Event:** year: 2012; month: 8; day: 26; eventRemarks: D.M. Takiya & A.P.M. Santos col.; **Record Level:** type: PhysicalObject; institutionCode: DZRJ; basisOfRecord: PreservedSpecimen

#### Distribution

Peru, Bolivia, Argentina.

**Distribution in Peru:** Junin, Cusco.

### Rhagovelia whitei

(Breddin, 1898)

#### Materials

- **Type status:** Other material. **Occurrence:** individualCount: 2; sex: 1 macropterous male, 1 apterous female; **Taxon:** genus: Rhagovelia; specificEpithet: whitei; **Location:** continent: South America; country: Brazil; stateProvince: Ceará; municipality: Ubajara; locality: Parque Nacional de Ubajara, Trilha Araticum, Rio das Minas; decimalLatitude: -3.83083; decimalLongitude: -40.90500; **Identification:** identifiedBy: F.F.F. Moreira; **Event:** year: 2014; month: 4; day: 23; eventRemarks: D. Takiya col.; **Record Level:** type: PhysicalObject; institutionCode: DZRJ; basisOfRecord: PreservedSpecimen
- **Type status:** Other material. **Occurrence:** individualCount: 12; sex: 5 apterous males, 1 macropterous male, 4 apterous females, 2 macropterous females; **Taxon:** genus: Rhagovelia; specificEpithet: whitei; **Location:** continent: South America; country: Brazil; stateProvince: Goiás; municipality: Cristalina; decimalLatitude: -16.730222; decimalLongitude: -47.569056; **Identification:** identifiedBy: F.F.F. Moreira; **Event:** year: 2013; month: 3; day: 19; eventRemarks: D.M. Takiya & A.P.M. Santos col.; **Record Level:** type: PhysicalObject; institutionCode: DZRJ; basisOfRecord: PreservedSpecimen
- **Type status:** Other material. **Occurrence:** individualCount: 7; sex: 1 apterous male, 6 apterous females; **Taxon:** genus: Rhagovelia; specificEpithet: whitei; **Location:** continent: South America; country: Brazil; stateProvince: Goiás; municipality: Alto Paraíso de Goiás; decimalLatitude: -14.144500; decimalLongitude: -47.482833; **Identification:** identifiedBy: F.F.F. Moreira; **Event:** year: 2013; month: 3; day: 23; eventRemarks: D.M. Takiya & A.P.M. Santos col.; **Record Level:** type: PhysicalObject; institutionCode: DZRJ; basisOfRecord: PreservedSpecimen
- **Type status:** Other material. **Occurrence:** individualCount: 5; sex: 1 apterous male, 4 apterous females; **Taxon:** genus: Rhagovelia; specificEpithet: whitei; **Location:** continent: South America; country: Brazil; stateProvince: Minas Gerais; municipality: Presidente Olegário; locality: Lapa da Fazenda São Bernardo; decimalLatitude: -18.28023; decimalLongitude: -46.11264; **Identification:** identifiedBy: F.F.F. Moreira; **Event:** year: 2013; month: 9; day: 30; eventRemarks: T. Zepon & L. Resende col.; **Record Level:** type: PhysicalObject; institutionCode: LES; basisOfRecord: PreservedSpecimen
- **Type status:** Other material. **Occurrence:** individualCount: 2; sex: 1 apterous male, 1 apterous female; **Taxon:** genus: Rhagovelia; specificEpithet: whitei; **Location:** continent: South America; country: Brazil; stateProvince: Minas Gerais; municipality: Presidente Olegário; locality: Lapa da Fazenda São Bernardo; decimalLatitude: -18.28023; decimalLongitude: -46.11264; **Identification:** identifiedBy: F.F.F. Moreira; **Event:** year: 2014; month: 4; day: 13; eventRemarks: T. Zepon & L. Resende col.; **Record Level:** type: PhysicalObject; institutionCode: LES; basisOfRecord: PreservedSpecimen

#### Distribution

Brazil, Paraguay.

**Distribution in Brazil:** PA, MA, CE!, MT, GO, MG, MS, SP.

### Rhagovelia zela

Drake, 1959

#### Materials

- **Type status:** Other material. **Occurrence:** individualCount: 6; sex: 3 apterous males, 3 apterous females; **Taxon:** genus: Rhagovelia; specificEpithet: zela; **Location:** continent: South America; country: Brazil; stateProvince: Goiás; municipality: Alto Paraíso de Goiás; decimalLatitude: -14.160972; decimalLongitude: -47.594167; **Identification:** identifiedBy: F.F.F. Moreira; **Event:** year: 2013; month: 3; day: 21; eventRemarks: D.M. Takiya & A.P.M. Santos col.; **Record Level:** type: PhysicalObject; institutionCode: DZRJ; basisOfRecord: PreservedSpecimen

#### Distribution

Brazil.

**Distribution in Brazil:** MT, GO!, MG, MS, ES, SP, RJ, SC.

### Stridulivelia strigosa

(Hungerford, 1929)

#### Materials

- **Type status:** Other material. **Occurrence:** individualCount: 1; sex: 1 brachypterous male; **Taxon:** genus: Stridulivelia; specificEpithet: strigosa; **Location:** continent: South America; country: Brazil; stateProvince: Amazonas; municipality: Manaus; locality: Bacia do Rio Cuieiras, Polícia Federal, igarapé de primeira ou segunda ordem; decimalLatitude: -2.70534; decimalLongitude: -60.38372; **Identification:** identifiedBy: F.F.F. Moreira; **Event:** year: 2004; month: 8; day: 19; eventRemarks: J. L. Nessimian & L. Fidelis col.; **Record Level:** type: PhysicalObject; institutionCode: DZRJ; basisOfRecord: PreservedSpecimen

#### Distribution

Venezuela, Guyana, Suriname, Brazil, Peru.

**Distribution in Brazil:** AP, PA, AM, MT.

### Stridulivelia transversa

(Hungerford, 1929)

#### Materials

- **Type status:** Other material. **Occurrence:** individualCount: 9; sex: 1 apterous male, 8 apterous females; **Taxon:** genus: Stridulivelia; specificEpithet: transversa; **Location:** continent: South America; country: Brazil; stateProvince: Amazonas; municipality: Manaus; locality: Bacia do Rio Cuieiras, tributário do Igarapé da Cachoeira; decimalLatitude: -2.69593; decimalLongitude: -60.29520; **Identification:** identifiedBy: F.F.F. Moreira; **Event:** year: 2004; month: 8; day: 20; eventRemarks: J. L. Nessimian & L. Fidelis col.; **Record Level:** type: PhysicalObject; institutionCode: DZRJ; basisOfRecord: PreservedSpecimen

#### Distribution

Suriname, Brazil.

**Distribution in Brazil:** AP, PA, AM.

### Nerthra terrestris

(Kevan, 1948)

#### Materials

- **Type status:** Other material. **Occurrence:** individualCount: 1; sex: 1 macropterous male; **Taxon:** genus: Nerthra; specificEpithet: terrestris; **Location:** continent: South America; country: Brazil; stateProvince: Pará; municipality: Altamira; locality: Abrigo da Gravura; decimalLatitude: -3.26614; decimalLongitude: -52.22215; **Identification:** identifiedBy: F.F.F. Moreira; **Event:** year: 2009; month: 7; day: 8; eventRemarks: M.E. Bichuette col.; **Record Level:** type: PhysicalObject; institutionCode: LES; basisOfRecord: PreservedSpecimen

#### Distribution

Colombia, Venezuela, Trinidad & Tobago, Guyana, Suriname, French Guiana, Brazil, Ecuador, Peru, Bolivia.

**Distribution in Brazil:** RR, PA!, AM, GO, MS, SP, RJ.

### Ambrysus stali

La Rivers, 1962

#### Materials

- **Type status:** Other material. **Occurrence:** individualCount: 1; sex: 1 macropterous male; **Taxon:** genus: Ambrysus; specificEpithet: stali; **Location:** continent: South America; country: Brazil; stateProvince: Pará; municipality: Altamira; locality: Carvena Kararaô; decimalLatitude: -3.13757; decimalLongitude: -51.82172; **Identification:** identifiedBy: F.F.F. Moreira; **Event:** year: 2009; month: 4; day: 10; eventRemarks: M. E. Bichuette, D. R. Pedroso, F. F. Pellegatti & T. L. C. Scatolini col.; **Record Level:** type: PhysicalObject; institutionCode: LES; basisOfRecord: PreservedSpecimen
- **Type status:** Other material. **Occurrence:** individualCount: 3; sex: 1 macropterous male, 2 macropterous females; **Taxon:** genus: Ambrysus; specificEpithet: stali; **Location:** continent: South America; country: Brazil; stateProvince: Pará; municipality: Altamira; locality: Caverna do Jacaré; decimalLatitude: -3.23346; decimalLongitude: -52.05397; **Identification:** identifiedBy: F.F.F. Moreira; **Event:** year: 2011; month: 4; day: 5; eventRemarks: M. E. Bichuette, J. E. Gallão, D. R. Pedroso & D. Monteiro-Neto col.; **Record Level:** type: PhysicalObject; institutionCode: LES; basisOfRecord: PreservedSpecimen

#### Distribution

Venezuela, Trinidad & Tobago, Suriname, French Guiana, Brazil, Argentina.

**Distribution in Brazil:** PA, AM.

### Limnocoris insignis

Stål, 1860

#### Materials

- **Type status:** Other material. **Occurrence:** individualCount: 3; sex: 2 macropterous males, 1 macropterous female; **Taxon:** genus: Limnocoris; specificEpithet: insignis; **Location:** continent: South America; country: Brazil; stateProvince: Rio de Janeiro; municipality: Rio de Janeiro; locality: Jacarepaguá, Taquara, Estrada Pau da Fome, Parque Estadual da Pedra Branca, Rio Figueira; decimalLatitude: -22.9327; decimalLongitude: -43.4380; **Identification:** identifiedBy: I.R.S. Cordeiro; **Event:** year: 2012; month: 10; day: 15; eventRemarks: I.R.S. Cordeiro & F.A.C. Silva col.; **Record Level:** type: PhysicalObject; institutionCode: DZRJ; basisOfRecord: PreservedSpecimen
- **Type status:** Other material. **Occurrence:** individualCount: 1; sex: 1 macropterous female; **Taxon:** genus: Limnocoris; specificEpithet: insignis; **Location:** continent: South America; country: Brazil; stateProvince: Rio de Janeiro; municipality: Rio de Janeiro; locality: Jacarepaguá, Taquara, Estrada Pau da Fome, Parque Estadual da Pedra Branca, Rio Figueira; decimalLatitude: -22.9327; decimalLongitude: -43.4380; **Identification:** identifiedBy: I.R.S. Cordeiro; **Event:** year: 2013; month: 6; day: 8; eventRemarks: I.R.S. Cordeiro, F.A.C. Silva, F.F. Silva, L. Chaves & A. Ferreira col.; **Record Level:** type: PhysicalObject; institutionCode: DZRJ; basisOfRecord: PreservedSpecimen
- **Type status:** Other material. **Occurrence:** individualCount: 5; sex: 4 macropterous males, 1 macropterous female; **Taxon:** genus: Limnocoris; specificEpithet: insignis; **Location:** continent: South America; country: Brazil; stateProvince: Rio de Janeiro; municipality: Rio de Janeiro; locality: Jacarepaguá, Taquara, Estrada Pau da Fome, Parque Estadual da Pedra Branca, Rio Figueira; decimalLatitude: -22.9327; decimalLongitude: -43.4380; **Identification:** identifiedBy: I.R.S. Cordeiro; **Event:** year: 2013; month: 8; day: 19; eventRemarks: I.R.S. Cordeiro, F.A.C. Silva, F.F. Silva, L. Chaves & A. Ferreira col.; **Record Level:** type: PhysicalObject; institutionCode: DZRJ; basisOfRecord: PreservedSpecimen
- **Type status:** Other material. **Occurrence:** individualCount: 1; sex: 1 macropterous male; **Taxon:** genus: Limnocoris; specificEpithet: insignis; **Location:** continent: South America; country: Brazil; stateProvince: Rio de Janeiro; municipality: Rio de Janeiro; locality: Jacarepaguá, Taquara, Estrada Pau da Fome, Parque Estadual da Pedra Branca, Rio Grande; decimalLatitude: -22.9320; decimalLongitude: -43.4452; **Identification:** identifiedBy: I.R.S. Cordeiro; **Event:** year: 2012; month: 11; day: 19; eventRemarks: I.R.S. Cordeiro & F.A.C. Silva col.; **Record Level:** type: PhysicalObject; institutionCode: DZRJ; basisOfRecord: PreservedSpecimen
- **Type status:** Other material. **Occurrence:** individualCount: 1; sex: 1 macropterous female; **Taxon:** genus: Limnocoris; specificEpithet: insignis; **Location:** continent: South America; country: Brazil; stateProvince: Rio de Janeiro; municipality: Rio de Janeiro; locality: Jacarepaguá, Taquara, Estrada Pau da Fome, Parque Estadual da Pedra Branca, Rio Grande; decimalLatitude: -22.9320; decimalLongitude: -43.4452; **Identification:** identifiedBy: I.R.S. Cordeiro; **Event:** year: 2013; month: 2; day: 23; eventRemarks: I.R.S. Cordeiro, F.A.C. Silva, F.F. Silva, L. Chaves & A. Ferreira col.; **Record Level:** type: PhysicalObject; institutionCode: DZRJ; basisOfRecord: PreservedSpecimen
- **Type status:** Other material. **Occurrence:** individualCount: 5; sex: 3 macropterous males, 2 macropterous females; **Taxon:** genus: Limnocoris; specificEpithet: insignis; **Location:** continent: South America; country: Brazil; stateProvince: Rio de Janeiro; municipality: Rio de Janeiro; locality: Jacarepaguá, Taquara, Estrada Pau da Fome, Parque Estadual da Pedra Branca, Rio Grande; decimalLatitude: -22.9320; decimalLongitude: -43.4452; **Identification:** identifiedBy: I.R.S. Cordeiro; **Event:** year: 2013; month: 5; day: 23; eventRemarks: I.R.S. Cordeiro, F.A.C. Silva, F.F. Silva, L. Chaves & A. Ferreira col.; **Record Level:** type: PhysicalObject; institutionCode: DZRJ; basisOfRecord: PreservedSpecimen
- **Type status:** Other material. **Occurrence:** individualCount: 28; sex: 8 macropterous males, 20 macropterous females; **Taxon:** genus: Limnocoris; specificEpithet: insignis; **Location:** continent: South America; country: Brazil; stateProvince: Rio de Janeiro; municipality: Rio de Janeiro; locality: Jacarepaguá, Taquara, Estrada Pau da Fome, Parque Estadual da Pedra Branca, Rio Grande; decimalLatitude: -22.9320; decimalLongitude: -43.4452; **Identification:** identifiedBy: I.R.S. Cordeiro; **Event:** year: 2014; month: 4; day: 19; eventRemarks: I.R.S. Cordeiro, F.A.C. Silva, F.F. Silva, L. Chaves, A. Ferreira, L.S. Barbosa, D.M.S. Corrêa & L.B. Pena Forte col.; **Record Level:** type: PhysicalObject; institutionCode: DZRJ; basisOfRecord: PreservedSpecimen

#### Distribution

Brazil.

**Distribution in Brazil:** MG, SP, RJ, PR, SC, RS.

### Enitharoides brasiliensis

(Spinola, 1837)

#### Materials

- **Type status:** Other material. **Occurrence:** individualCount: 1; sex: 1 coleopteroid female; **Taxon:** genus: Enitharoides; specificEpithet: brasiliensis; **Location:** continent: South America; country: Brazil; stateProvince: Rio de Janeiro; municipality: Rio de Janeiro; locality: Jacarepaguá, Taquara, Estrada Pau da Fome, Parque Estadual da Pedra Branca, Rio Grande; decimalLatitude: -22.9320; decimalLongitude: -43.4452; **Identification:** identifiedBy: I.R.S. Cordeiro; **Event:** year: 2013; month: 2; day: 23; eventRemarks: I.R.S. Cordeiro, F.A.C. Silva, F.F. Silva, L. Chaves & A. Ferreira col.; **Record Level:** type: PhysicalObject; institutionCode: DZRJ; basisOfRecord: PreservedSpecimen
- **Type status:** Other material. **Occurrence:** individualCount: 1; sex: 1 coleopteroid male; **Taxon:** genus: Enitharoides; specificEpithet: brasiliensis; **Location:** continent: South America; country: Brazil; stateProvince: Rio de Janeiro; municipality: Rio de Janeiro; locality: Jacarepaguá, Taquara, Estrada Pau da Fome, Parque Estadual da Pedra Branca, Rio Grande; decimalLatitude: -22.9320; decimalLongitude: -43.4452; **Identification:** identifiedBy: I.R.S. Cordeiro; **Event:** year: 2013; month: 9; day: 7; eventRemarks: I.R.S. Cordeiro, F.A.C. Silva, F.F. Silva, L. Chaves & A. Ferreira col.; **Record Level:** type: PhysicalObject; institutionCode: DZRJ; basisOfRecord: PreservedSpecimen

#### Distribution

Brazil.

**Distribution in Brazil:** MG, ES, RJ.

### Martarega bentoi

Truxal, 1949

#### Materials

- **Type status:** Other material. **Occurrence:** individualCount: 1; sex: 1 brachypterous male; **Taxon:** genus: Martarega; specificEpithet: bentoi; **Location:** continent: South America; country: Brazil; stateProvince: Rio de Janeiro; municipality: Rio de Janeiro; locality: Jacarepaguá, Taquara, Estrada Pau da Fome, Parque Estadual da Pedra Branca, Rio Grande; decimalLatitude: -22.9320; decimalLongitude: -43.4452; **Identification:** identifiedBy: I.R.S. Cordeiro; **Event:** year: 2014; month: 4; day: 19; eventRemarks: I.R.S. Cordeiro, F.A.C. Silva, F.F. Silva, L. Chaves, A. Ferreira; L.S. Barbosa, D.M.S. Corrêa & L.B. Pena Forte; **Record Level:** type: PhysicalObject; institutionCode: DZRJ; basisOfRecord: PreservedSpecimen

#### Distribution

Brazil, Argentina.

**Distribution in Brazil:** PI, PE, MT, MG, RJ!.

## Discussion

 Some of the records above form the first inventory of aquatic Heteroptera from Pedra Branca State Park, Rio de Janeiro, Brazil (Cordeiro 2015). No water bugs had been previously recorded from the area, and the following species have been collected in four rivers inside the park: *Brachymetra albinervis albinervis*, *B. furva*, *Rhagovelia itatiaiana*, *R. lucida*, *R. macta*, *Limnocoris insignis*, *Enitharoides brasiliensis*, and *Martarega bentoi*. Among the records from this park, the occurrence of two species pairs is very interesting: *B. albinervis albinervis* / *B. furva* and *R. itatiaiana* / *R. macta*. Each pair of species are morphologically very similar, differing mainly in structures exclusive to males, which may lead to misidentifications (e.g. *R. macta* in Moreira and Ribeiro 2009), especially because they might co-occur in their geographical ranges.

Additional material collected in other Brazilian national or state parks was also examined and resulted in several first records of aquatic Heteroptera from each park. These include: *Halobatopsis platensis*, *Hydrometra fruhstorferi* and *Rhagovelia janeira* from Iguaçu National Park, *Limnogonus profugus* and *R. whitei* from Ubajara National Park, and *Neogerris lubricus*, *Microvelia ayacuchana*, *M. pulchella* and *Platyvelia brachialis* from Sete Cidades National Park. The exceptions are *Rheumatobates minutus flavidus* and *Microvelia venustatis* from Serra do Mar State Park, and *Rhagovelia accedens*, *R. aiuruoca* and *R. triangula* from Caparaó National Park, areas where other species of Gerromorpha and Nepomorpha had already been recorded (e.g. Moreira et al. 2010, Moreira and Barbosa 2011). Such efforts are useful to expand the parks’ lists of species, which is important to reinforce their value as conservation areas.

Some of the records also serve to fill gaps on the known geographical distributions of species that occur throughout wide geographical ranges, but had never been collected in some areas within it. That is the case of *Halobatopsis platensis* and *Martarega bentoi*, known from northeastern Brazil to Argentina, but new respectively to Goiás and Rio de Janeiro; *Halobatopsis spiniventris*, *Hydrometra fruhstorferi* and *Rhagovelia janeira*, from southeastern Brazil to Argentina, but new to Paraná; *Neogerris lubricus*, common throughout South America, new to Piauí; *Rheumatobates minutus flavidus*, from Costa Rica to Argentina, new to São Paulo; *Mesovelia amoena*, *Microvelia pulchella* and *Platyvelia brachialis*, from North America to Argentina, new to Bahia and Piauí; *Rhagovelia whitei*, from northern Brazil to Paraguay, new to Ceará; and *Nerthra terrestris*, from Colombia to southeastern Brazil, new to Pará. Finally, distribution expansions include northernmost records of *Limnogonus profugus*, *Rhagovelia lucida* and *R. zela* (Ceará/ Espírito Santo/ Goiás), the first record of *Rheumatobates bonariensis* from central-western Brazil (Mato Grosso), and the first record of *Microvelia ayacuchana* from northeastern Brazil (Piauí).

## Supplementary Material

XML Treatment for Brachymetra albinervis albinervis

XML Treatment for Brachymetra furva

XML Treatment for Halobatopsis platensis

XML Treatment for Halobatopsis spiniventris

XML Treatment for Limnogonus profugus

XML Treatment for Neogerris lubricus

XML Treatment for Potamobates spiculus

XML Treatment for Rheumatobates bonariensis

XML Treatment for Rheumatobates minutus flavidus

XML Treatment for Hydrometra fruhstorferi

XML Treatment for Mesovelia amoena

XML Treatment for Mesovelia mulsanti

XML Treatment for Euvelia lata

XML Treatment for Microvelia ayacuchana

XML Treatment for Microvelia longipes

XML Treatment for Microvelia mimula

XML Treatment for Microvelia pulchella

XML Treatment for Microvelia summersi

XML Treatment for Microvelia ubatuba

XML Treatment for Microvelia urucara

XML Treatment for Microvelia venustatis

XML Treatment for Platyvelia brachialis

XML Treatment for Rhagovelia accedens

XML Treatment for Rhagovelia aiuruoca

XML Treatment for Rhagovelia elegans

XML Treatment for Rhagovelia fontanalis

XML Treatment for Rhagovelia itatiaiana

XML Treatment for Rhagovelia janeira

XML Treatment for Rhagovelia lucida

XML Treatment for Rhagovelia macta

XML Treatment for Rhagovelia traili

XML Treatment for Rhagovelia triangula

XML Treatment for Rhagovelia trianguloides

XML Treatment for Rhagovelia versuta

XML Treatment for Rhagovelia whitei

XML Treatment for Rhagovelia zela

XML Treatment for Stridulivelia strigosa

XML Treatment for Stridulivelia transversa

XML Treatment for Nerthra terrestris

XML Treatment for Ambrysus stali

XML Treatment for Limnocoris insignis

XML Treatment for Enitharoides brasiliensis

XML Treatment for Martarega bentoi
